# Ocular metastasis in breast cancer: management of clinical case reports and a literature review

**DOI:** 10.3389/fonc.2025.1605881

**Published:** 2026-01-14

**Authors:** Jizhuo Gao, Qian Cao, Yi Liu, Mengyao Huang, Man Li, Lingzhi Xu

**Affiliations:** 1 Department of Oncology, The Second Affiliated Hospital of Dalian Medical University, Dalian, Liaoning, China; 2 Department of Radiology, The Second Affiliated Hospital of Dalian Medical University, Dalian, Liaoning, China

**Keywords:** case report, diagnosis, metastatic breast cancer, ocular metastasis, prognosis

## Abstract

In recent years, the incidence of breast cancer (BC) and its distant metastases, including ocular metastasis (OM), has continued to rise. However, reports on BC-OM remain scarce. OM is rare and often asymptomatic. It can be easily masked by metastases in other organs, increasing the risk of misdiagnosis, and thus warrants greater clinical attention. In this paper, we describe three cases of BC-OM, review its epidemiological and clinical features, and summarize diagnostic methods and treatment strategies. Our aim is to provide a reference for clinical practice, support standardized management, and ultimately improve patient survival and quality of life (QoL).

## 1 Introduction

According to the latest global cancer statistics (1), breast cancer (BC) is the most common malignancy in women and the leading cause of death among those aged 40–79 years. With improved screening, advances in diagnostic techniques, and the introduction of novel therapies, progression-free survival (PFS) and overall survival (OS) in BC have increased markedly, along with the detection of metastases, including the rare ocular metastases (OM).

BC-OM is typically asymptomatic, and can be easily obscured by metastases in other organs or treatment-related toxicities. Although not immediately life-threatening, OM can impair vision and significantly compromise quality of life (QoL), which warrants greater clinical attention.

Here, we retrospectively analyzed three BC-OM patients at our center: one hormone receptor-positive BC and two triple-negative BC. One patient presented with OM at the initial diagnosis of advanced disease, while the other two developed OM during disease progression after multiple lines of therapy. We further summarized our experience together with relevant literature to enhance understanding of BC-OM and provide a reference for future clinical practice.

## 2 Case report

### 2.1 Case 1

**Figure 1:**
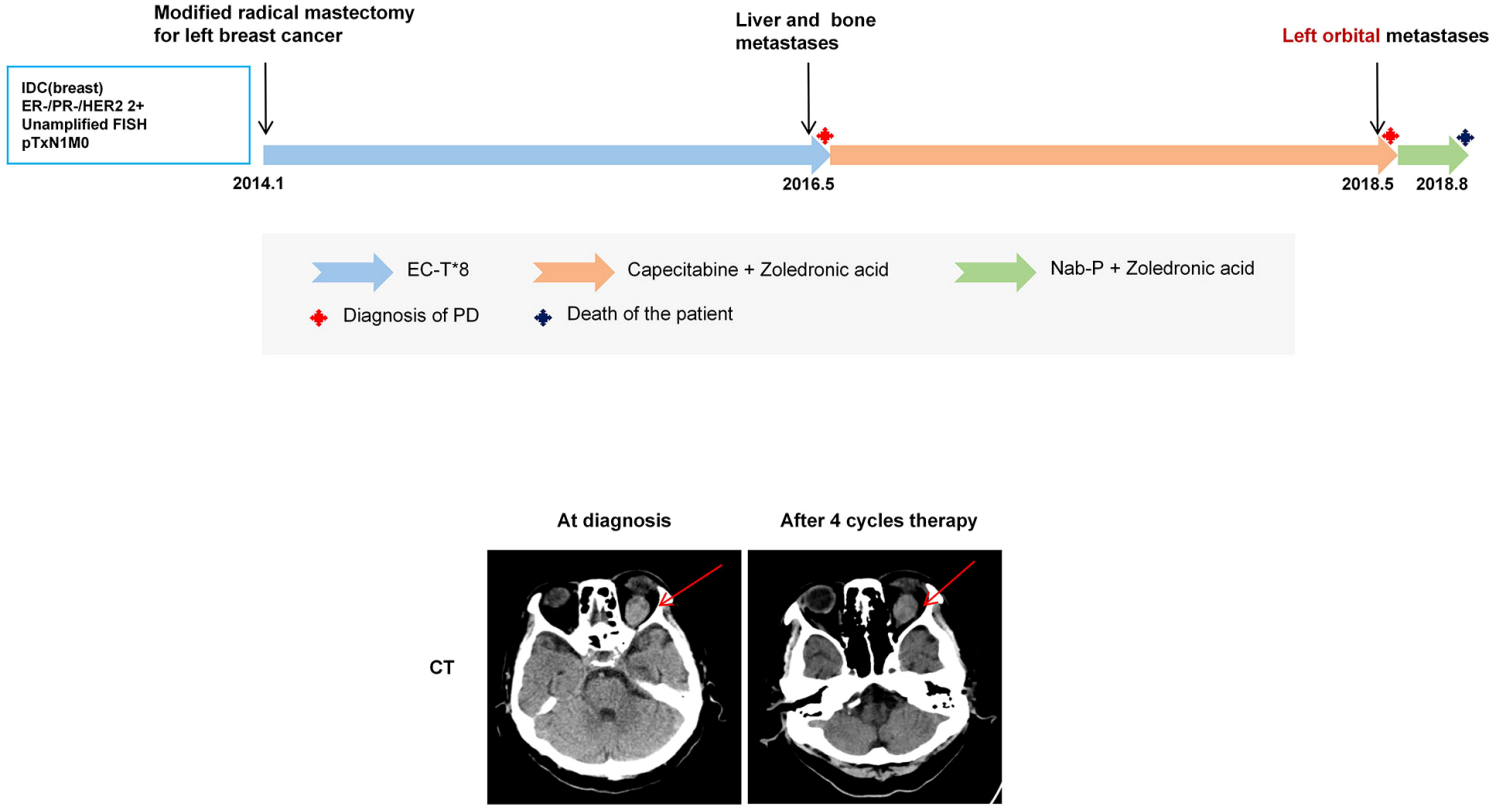
The upper panel shows the treatment timeline of Patient A. The lower panel shows CT evaluation of the left orbital lesion at the time of ocular metastasis diagnosis and after four cycles of Nab-p treatment. A high-density mass was identified in the left retrobulbar superior rectus muscle region (red arrow), with the maximum diameter reduced from approximately 1.7 × 2.5 cm to 1.6 × 2.4 cm.

Patient A, a 71-year-old female, with no significant comorbidities or family history, was diagnosed in January 2014 with invasive ductal carcinoma of the left breast and underwent a left modified radical mastectomy (pTxN1M0). Immunohistochemistry (IHC) showed ER (-), PR (-), HER2 (2+, FISH-), and Ki-67 (30%+). She received postoperative epirubicin–cyclophosphamide followed by docetaxel (EC-T) chemotherapy and switched to capecitabine in May 2016 after recurrence with liver and bone metastases. In May 2018, she developed decreased vision, diplopia, and swelling of the left eye, and an ocular computed tomography (CT) suggested left orbital metastasis of BC. After the patient was switched to nanoparticle albumin-bound-paclitaxel (Nab-p) for four cycles of treatment, the ocular discomfort improved, and the lesion shrank slightly. However, the patient died three months later due to systemic complications including anemia, hypoproteinemia, infection, and cancer pain (**Figure 1**).

The patient initially presented with ocular symptoms, subsequently confirmed as OM on imaging. Although systemic Nab-p achieved transient symptom relief, disease progression with severe complications led to death, with an OS of 55 months and an OM-specific survival of only 3 months. Local ocular therapy was withheld due to poor performance status and extensive systemic involvement. This case illustrates that in heavily pretreated advanced breast cancer, OM may indicate rapid deterioration and a limited therapeutic window. Moreover, visual disturbances in such patients should not be attributed solely to chemotherapy-related neurotoxicity, and OM should be carefully considered.

### 2.2 Case 2

**Figure 2:**
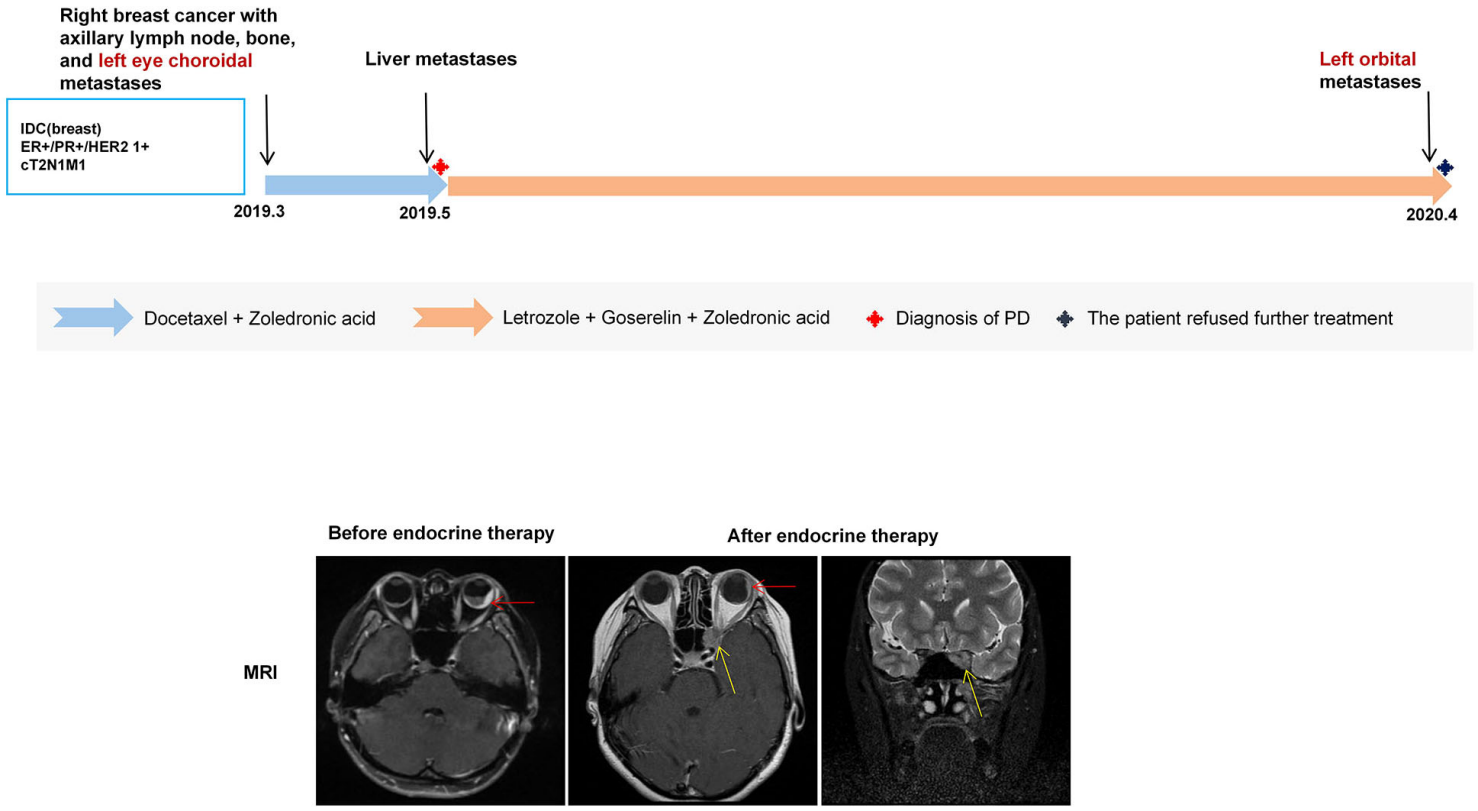
The upper panel shows the treatment timeline of Patient B. The lower panel shows pre- and post-endocrine therapy brain MRI findings. MRI demonstrates irregular thickening of the bilateral ocular rings with marked enhancement, particularly at the posterior wall of the left ocular ring. After therapy, the left eye choroidal lesion (red arrow) decreased from 5 mm to 3 mm. A new T2-weighted slightly hyperintense lesion at the left orbital apex involving the sphenoid sinus and optic nerve was also observed (yellow arrow), with a maximum diameter of approximately 10 × 8 mm.

Patient B, a 41-year-old female without relevant comorbidities or family history, presented in March 2019 with blurred and distorted vision in the left eye. Positron emission tomography–computed tomography (PET-CT) revealed right BC with right axillary lymph node and bone metastases, as well as left choroidal metastasis. Biopsy confirmed invasive ductal carcinoma of the right breast (cT2N1M1, stage IV) with IHC showing ER (90%+), PR (80%+), HER2 (1+), and Ki-67 (50%+). She initially received docetaxel with zoledronic acid during which hepatic metastases developed while the ocular lesion remained stable. Subsequent treatment with letrozole plus ovarian function suppression (OFS) led to shrinkage of the choroidal lesion and relief of ocular symptoms. In April 2020, new orbital metastasis appeared in the left eye and the patient declined further treatment (**Figure 2**).

This case illustrates that ocular metastasis may present as an initial manifestation of BC and warrants clinical attention. The patient achieved a PFS of approximately 11 months for the choroidal metastasis and an OS of at least 13 months after diagnosis of BC with OM. Local ocular therapy was not considered because systemic endocrine therapy effectively controlled ocular symptoms. However, ipsilateral orbital metastases emerged despite well-controlled choroidal lesions, suggesting that OM at different anatomical sites may exhibit varying sensitivities to oncological treatments due to tumor heterogeneity.

### 2.3 Case 3

**Figure 3:**
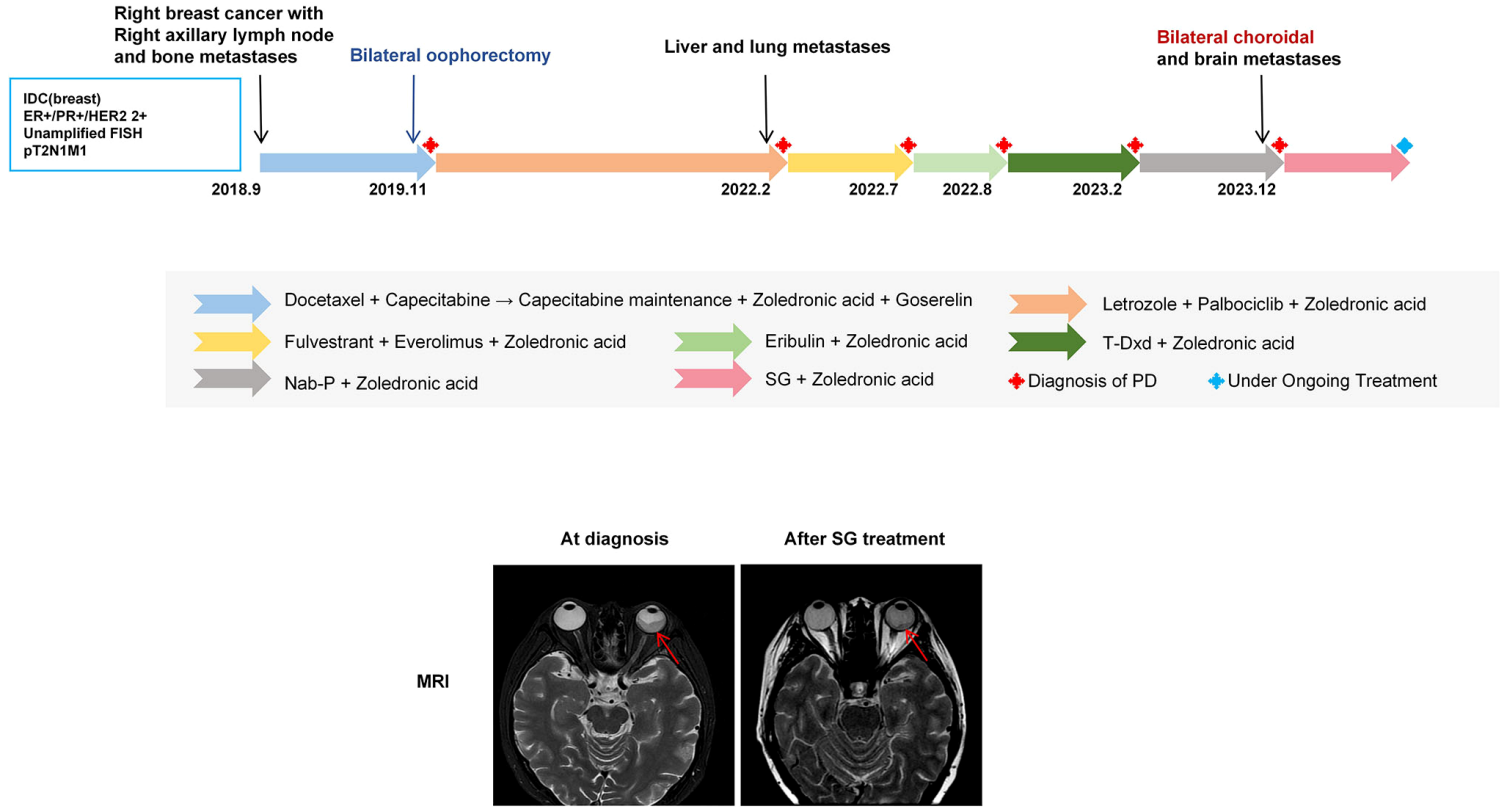
The upper panel shows the treatment timeline of Patient C. The lower panel shows pre- and post-sacituzumab govitecan (SG) brain MRI findings. MRI reveals thickening posterior to the left ocular ring with patchy and nodular enhancement on contrast-enhanced imaging. After SG treatment, the thickest part of the lesion (red arrow) decreased from 8.3 mm to 4.7 mm.

Patient C, a 46-year-old female with no significant comorbidities or family history, was diagnosed in September 2018 with right BC involving axillary lymph nodes and bone metastases (cT2N2M1, stage IV). IHC revealed ER (90%+), PR (1%+), HER2 (2+, FISH-), and Ki-67 (15%+). She initially received four cycles of docetaxel plus capecitabine followed by capecitabine maintenance therapy. In November 2019, the patient was switched to letrozole + OFS combined with capecitabine after progressive disease (PD). In February 2022, she presented with new bilateral lung metastases and hepatic metastases and was switched to fluvastatin + everolimus, eribulin, trastuzumab deruxtecan (T-Dxd), and Nab-p. In December 2023, she presented with bilateral choroidal metastases, severe in the left eye with vision loss and an exophytic mass, accompanied by newly detected brain metastases. Treatment with sacituzumab govitecan (SG) stabilized ocular lesions, though central nervous system (CNS) disease progressed (**Figure 3**).

Unlike typical presentations, this patient’s OM manifested as an exophytic mass with atypical morphology. Rapid tumor growth within the restricted intraocular space led to early vision loss, necessitating prompt intervention. This case underscores the importance of early detection and demonstrates that timely treatment modification can effectively control ocular symptoms. Moreover, Patient C developed brain metastases shortly after OM diagnosis, suggesting a potential association between the biological behavior of OM and subsequent brain metastases.

## 3 Discussion

### 3.1 Overview of breast cancer ocular metastases

#### 3.1.1 Epidemiology

BC is the most common malignancy in women (1) and accounts for nearly 49% of OM originating from BC (2). Although OM primarily spreads via the hematogenous route, its incidence remains low (0.5%–10%) (3, 4), likely due to the protective effects of the blood-aqueous and blood-retinal barriers. About 30% of patients present with OM at initial diagnosis, whereas nearly 50% develop it 20–40 months after surgery—later than in most other malignancies (5). OM occurs more frequently in the left eye, probably because tumor emboli from the left common carotid artery reach the left ophthalmic artery more directly, whereas emboli from the right must bypass the innominate artery. All three patients in this study had left-eye involvement, consistent with this pattern. Histopathological studies further indicate that invasive lobular carcinoma (ILC) has a higher propensity for ocular spread than invasive ductal carcinoma (IDC), possibly attributed to the E-cadherin-mediated restriction of cell dissemination in IDC (6).

#### 3.1.2 Anatomical features of ocular metastasis

BC-OM includes intraocular and orbital metastases. Intraocular metastasis mainly involves the choroid, iris, ciliary body, and retina, among which the choroid is the preferred site of OM due to its rich blood supply, extensive blood vessels, and slow blood flow (2, 3, 7). Choroidal metastases account for about 81% of all OM, primarily found in the posterior pole of the eye, optic papilla, and peripapillary macula, which is closely related to the distribution of choroidal blood vessels. Iris metastases account for 9%, optic disk for 5%, ciliary body for 2%, and retinal metastases for less than 1% (8). Orbital metastases are less common, accounting for about 3% to 10% (9, 10), and may involve orbital bone, fat, muscle, nerves, and blood vessels.

### 3.2 Clinical manifestations

BC-OM symptoms include proptosis, restricted eye movement, diplopia, eyelid swelling, vision loss, conjunctival congestion, blurred vision, eye pain, and eye invagination. Some patients present with a palpable eyelid mass (11), but most seek medical attention following sudden vision loss. Rapid progression can result in near-total vision loss, whereas 7% remain initially asymptomatic (12).

Clinical manifestations vary with metastatic site. Choroidal metastases, often involving the macula, typically cause rapid visual decline due to macular damage, subretinal fluid, retinal detachment, or hyperopia, enabling early detection (13). In this study, all three choroidal metastasis cases presented with blurred vision, consistent with previous reports. Orbital metastases commonly induce ocular pain and periorbital masses due to tumor traction, leading to globe invagination or motility restriction (14). Ocular pain may arise from inflammation, tumor necrosis, glaucoma, scleral involvement, or ciliary nerve compression (15, 16). One patient in this study with orbital metastasis reported ocular pain, in line with the literature. Conjunctival and optic nerve canal metastases may manifest as painless conjunctival masses or progressive vision loss (17, 18). Involvement of extraocular muscles or the oculomotor nerve can result in strabismus, paralysis, or restricted movement.

### 3.3 Relationship to other metastases

BC-OM is typically unilateral but may progress to bilateral, multifocal involvement alongside systemic metastases in later stages (3, 19, 20). While some BC cases are initially identified due to OM symptoms, most OM cases occur secondary to visceral, bone, or CNS metastases. In this study, one of three patients (33.3%) presented with OM as the initial diagnosis, consistent with previous reports. Asymptomatic choroidal metastases occur in 5% of metastatic BC cases, increasing to 11% in multiorgan metastases, with 67% of detected alongside involvement of other organs (21), suggesting that multiorgan metastasis as a high-risk factor for OM.

Brain metastases occur in approximately 6% of BC cases but rise to 60% among patients with OM (4). Kuhn et al. proposed that cancer cells metastasize to the choroid and spread to the brain via the optic nerve after interacting with pigmented cells (22). Choroidal metastases more frequently lead to brain involvement than orbital metastases, potentially worsening prognosis. Timely cranial CT or MRI upon BC-OM diagnosis is recommended for early detection, optimized treatment, and improved outcomes. In this study, patient C developed brain (meningeal) metastases shortly after or concurrently with OM, consistent with literature findings.

### 3.4 Diagnosis

Asymptomatic or mildly symptomatic OM patients often neglect regular eye examinations, resulting in missed or delayed diagnoses. Early detection and intervention are essential for preserving visual function and improving QoL. BC-OM diagnosis requires integrating patient history, clinical signs, and auxiliary tests, including ophthalmic imaging, general imaging, and pathological biopsy. Ocular ultrasound serves as an initial screening tool, while biopsy remains the gold standard but requires strict indications. Comprehensive evaluation supports accurate diagnosis, lesion localization, and treatment planning.

#### 3.4.1 Common ophthalmologic imaging

Fundoscopy and slit-lamp examination are primary methods for detecting lesions in the sclera, anterior chamber, lens, and retina, making them preferred initial assessments (17, 18). Retinal metastases typically appear as yellowish-white swellings with subretinal fluid or hemorrhage (23). Optic disc metastases may present as thickened papillae or nodules (24), while iris metastases appear as yellow or white nodules under slit-lamp examination. Fluorescein fundus angiography (FFA) is a key diagnostic tool for choroidal metastases, using sodium fluorescein to highlight lesions. Early-stage metastases appear as venous non-fluorescent areas, while later-stage masses exhibit strong fluorescence with well-defined borders. Rapid tumor growth may lead to tissue necrosis, causing extensive low-fluorescence areas. For patients unable to tolerate angiography, fundus autofluorescence (FAF) offers a noninvasive alternative, showing fluorescence deficits at lesions with granular hyperfluorescence at the edges (25). Optical coherence tomography (OCT) effectively visualizes retinal thickening and detachment.

#### 3.4.2 Other imaging tests

Ultrasound detects moderate to high-intensity echoes (26) and abundant blood flow, aiding both screening and treatment evaluation. It is particularly useful when severe retinopathy causes vitreous hemorrhage, which can obstruct retinal assessment via funduscopy or fluorescein angiography. Computed tomography (CT) primarily evaluates orbital bone involvement. In choroidal metastases, CT typically shows flattened soft tissue masses of uneven density or irregular thickening of the ocular ring. In orbital metastases, it may reveal dense periorbital soft tissue masses, muscle thickening, or swelling, with or without bone destruction. Magnetic resonance imaging (MRI) offers superior soft tissue visualization and is not affected by bleeding. Choroidal metastases generally appear as low or isointense signals on T1-weighted images and hyperintense signals on T2-weighted images, with patchy hyperintense areas in acute hemorrhage. MRI also helps differentiate choroidal melanoma, which usually exhibits a high signal on T1-weighted images (27).

Combining CT and MRI is often sufficient for diagnosing and localizing ocular metastases (28). However, systemic evaluation is essential to identify the primary tumor and other metastases. PET-CT helps assess systemic disease and guide treatment. Ferdinand N et al. reported a case where PET-CT confirmed prostate cancer metastasis to the eye via prostate-specific membrane antigen (PSMA) expression, avoiding invasive diagnostic procedures (29).

#### 3.4.3 Intraocular pathology biopsy

Histopathologic biopsy remains the gold standard for diagnosing BC-OM, particularly when the primary tumor is unclear. It allows differentiation of immunohistochemical profiles between primary and metastatic lesions, aiding treatment planning and response prediction (30). Biopsy is typically reserved for isolated orbital metastases without systemic spread. Fine-needle aspiration biopsy (FNAB) has improved diagnostic accuracy, reducing the false-negative rate to less than 3% and the risk of retinal detachment to less than 4% for intraocular metastases thicker than 2 mm (31). Advances such as the vitrectomy knife have enabled minimally invasive sampling (32).

Despite its value, biopsy carries risks, including intraocular hemorrhage, tumor spread, and vision loss. In patients with a known primary tumor and multiple metastases, careful risk-benefit assessment is essential (33). In this study, all three patients were diagnosed based on BC history, symptoms, and imaging, thereby avoiding unnecessary biopsies and associated risks.

### 3.5 Treatment

Ocular metastatic cancer typically occurs in advanced malignancies with systemic, multiorgan involvement, making curative surgery impractical. Treatment primarily focuses on symptom relief. Since metastatic lesions share biological characteristics with the primary tumor, standardized systemic therapy can help control ocular involvement (34–36). Currently, no standardized protocol exists for BC-OM, highlighting the need for multidisciplinary collaboration. Treatment decisions are guided by metastasis burden, visual function, and life expectancy. Systemic therapy remains the cornerstone of management, often supplemented by local treatments to improve survival and QoL (37).

#### 3.5.1 Systemic therapy

BC patients with OM often have metastases to other organs, and OM generally responds to systemic therapy in a manner similar to other metastatic sites (19, 38, 39). Therefore, treatment selection should be guided by molecular subtyping and may include chemotherapy, endocrine therapy (40–42), targeted therapy (43, 44), and immunotherapy (45). However, the choroidal microenvironment exhibits high Fas ligand and PD-L1 expression with low CD8+ T cell infiltration, which may limit the effectiveness of immunotherapy (46, 47).

Studies have shown that approximately 80% of OM cases improve or stabilize with systemic therapy, although 53% of patients develop new OM during treatment (37). For example, Patient B in this study experienced regression of choroidal lesions but developed new orbital metastases after a period of letrozole-based endocrine therapy. This may be related to the choroid’s rich blood supply and slow blood flow, which could facilitate anticancer drug penetration and achieve adequate therapeutic concentrations (48).

#### 3.5.2 Localized treatment

Local treatments induce local inflammation, leading to cell death, vascular thrombosis, and autophagy, making them suitable for patients who are refractory or intolerant to systemic therapy, or those requiring rapid symptom relief (49). These treatments include radiation therapy, laser therapy, photodynamic therapy, local injections, and surgery.

Ocular metastases respond well to radiation therapy, primarily external beam radiation therapy (EBRT) and proton beam therapy, with the choice depending on lesion size and prognosis. EBRT, the most commonly used modality, typically delivers 30Gy/15Fx (4), achieving lesion shrinkage in 63%–83% of cases with a 63%–84% response rate (20, 50). However, prolonged survival increases the risks of ocular erythema, conjunctivitis, and cataracts (51). Proton beam therapy provides more precise irradiation, achieving an 84% response rate with a 29% lower complication rate than EBRT (50, 52). Laser therapy, particularly transpupillary thermotherapy (TTT), is effective for choroidal metastases. It uses semiconductor laser-induced thermal energy to induce vascular occlusion and tumor necrosis. TTT is safe, especially for small or early-stage lesions, and helps preserve vision. Photodynamic therapy (PDT), often combined with systemic treatment (33), destroys tumors through photosensitizer activation, resulting in cytotoxicity, vascular damage, and immune response stimulation (53). A small study (54) reported 78% effectiveness in choroidal metastases. However, PDT requires Verteporfin injection and maintaining an upright posture during treatment, posing risks such as acute vision loss and requiring further refinement. Intravitreal bevacizumab injection (55, 56) reduces metastatic foci and improves vision by inhibiting vascular endothelial growth factor (VEGF), but is ineffective without systemic therapy and is not recommended as a primary treatment (57). Surgical removal is reserved for cases with uncertain diagnoses, extensive invasion, intractable pain, or blindness. It is palliative, aiming for symptom relief rather than cure. Currently, no guidelines exist for selecting or timing local treatments, highlighting the need for further research. In our cases, some achieved local control with systemic therapy, while others deteriorated too rapidly to benefit from local intervention.

### 3.6 Regression and prognosis

Ocular metastatic cancer represents an advanced malignancy that often involves multiple organs. Although visual prognosis is generally favorable after treatment, systemic outcomes remain poor and vary depending on the primary tumor and type of metastasis (33). The presence of ocular metastasis further worsens prognosis, with median survival of BC patients ranging from 5 to 17 months (19). Advances in anticancer drugs and radiotherapy have improved survival and QoL in BC-OM patients, highlighting the importance of early detection, proactive treatment, and prevention of multi-organ metastasis.

## 4 Summary

**Figure 4:**
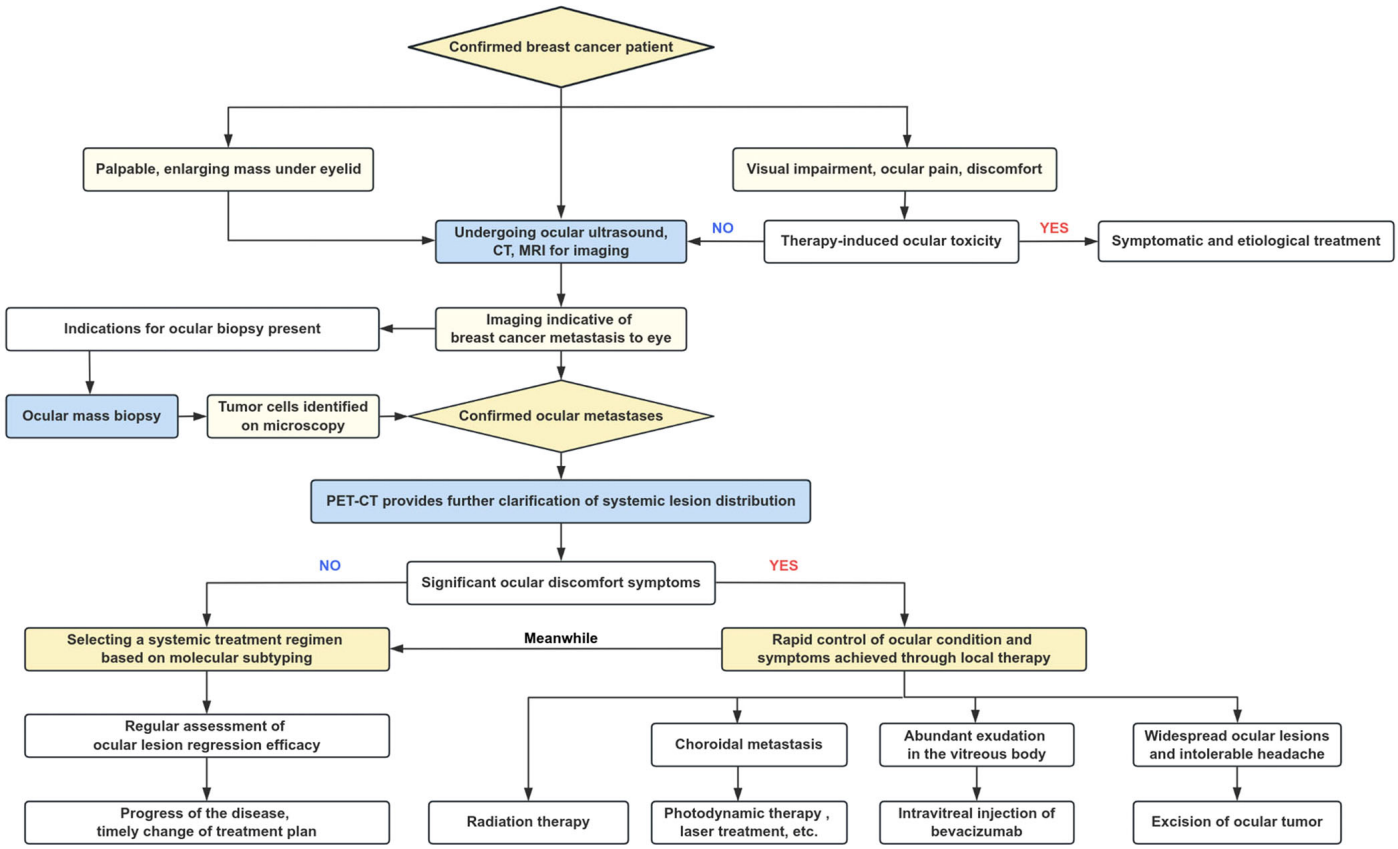
Proposed diagnostic and therapeutic pathway for BC-OM.

The three patients in this study progressed from initial consultation to a diagnosis of OM, reflecting heightened clinical awareness, advances in BC treatment, and the value of dynamic monitoring. Based on our center’s experience and this review of the literature, we propose a diagnostic and therapeutic reference for BC-OM (**Figure 4**). Ophthalmologic screening should be reinforced, particularly in asymptomatic BC patients, through regular visual acuity assessments, slit-lamp examinations, and ocular ultrasound to facilitate early detection. Symptomatic patients require prompt ophthalmologic evaluation, integrating medical history, imaging, and biopsy to confirm OM and distinguish it from systemic therapy-related side effects or other ocular conditions. Once diagnosed, systemic therapy remains the cornerstone of management, supplemented by local radiotherapy to improve QoL and potentially extend survival. Lesion changes should be dynamically monitored throughout treatment. Although BC-OM is associated with a poor prognosis, increased awareness, early intervention, and regular follow-up can slow disease progression, improve outcomes, and enhance patient QoL.

## Data Availability

The original contributions presented in the study are included in the article/supplementary material. Further inquiries can be directed to the corresponding authors.
